# Use a web-app to improve breast cancer risk factors and symptoms knowledge and adherence to healthy diet and physical activity in women without breast cancer diagnosis (Precam project)

**DOI:** 10.1007/s10552-022-01647-x

**Published:** 2022-11-08

**Authors:** Rubén Martín-Payo, Andrea Martínez-Urquijo, Edurne Zabaleta-del-Olmo, María del Mar Fernandez-Alvarez

**Affiliations:** 1 Facultad de Medicina y Ciencias de la Salud, Universidad de Oviedo, Oviedo, Spain; 2 Precam Research Group, Instituto de Investigación Sanitaria del Principado de Asturias (ISPA), Oviedo, Spain; 3 Hospital de Cruz Roja, Gijón, Spain; 4 Fundació Institut Universitari per a la recerca a l’Atenció Primària de Salut Jordi Gol i Gurina (IDIAPJGol), Barcelona, Spain; 5 Gerència Territorial de Barcelona, Institut Català de la Salut, Barcelona, Spain; 6 Department of Nursing, Faculty of Nursing, Universitat de Girona, Girona, Spain

**Keywords:** Breast neoplasm, Health promotion, Computer communication networks, Telemedicine

## Abstract

**Purpose:**

This study aimed to evaluate the preliminary effectiveness of an educational intervention using a web-app to improve knowledge of breast cancer risk factors and symptoms and adherence to healthy eating and physical activity among women without breast cancer diagnosis in Asturias (Spain).

**Methods:**

A pragmatic randomized pilot trial was conducted to evaluate the impact of a web-app-based intervention for women without breast cancer diagnosis. Women in the intervention group participated in a 6-month intervention web-app based on the Behaviour Change Wheel Model. The web-app includes information about breast cancer risk factors, early detection, physical activity and diet.

**Results:**

Two hundred and eighty-fifth women aged 25–50 were invited to join the study. Two hundred and twenty-four were randomly assigned to either the intervention group (IG = 134) or control group (CG = 90) according to their place of residence. Adherence among women in the IG increased significantly from pre- to post-intervention for eight of the 12 healthy behaviors and for the identification of six risk factors and six symptoms compared to women in the CG and, among whom adherence only increased for two behaviors, the identification of one risk factor and 0 symptoms. The intervention significantly improved the mean number of risk factors + 1.06 (*p* < 0.001) and symptoms + 1.18 (*p* < 0.001) identified by women in the IG.

**Conclusions:**

The preliminary results of this study suggest that an educational intervention using a web-app and based on the Behaviour Change Wheel model could be useful to improve knowledge of breast cancer risk factors and symptoms and to improve adherence to a healthy diet and physical activity in women without a previous breast cancer diagnosis.

**Supplementary Information:**

The online version contains supplementary material available at 10.1007/s10552-022-01647-x.

## Introduction

Breast cancer is one of the most common tumors in women and the most frequent in women under 40 years of age [1]. Approximately 33,000 cases of this type of cancer are diagnosed annually in Spain [2].

Some risk factors associated with the development of breast cancer, such as age, family history, and age at menarche and menopause, among others, are considered non-modifiable [3]. However, a number of risk factors can be influenced and therefore eliminated depending on their nature. In young women specifically, there is evidence of an association between breast cancer and unhealthy diets, sedentary lifestyles, and obesity [4, 5].

According to Borgquist et al. [6], there are several clinical challenges involved in breast cancer prevention, including the development of prevention programs targeting individual risk factors. These prevention programs should not be limited to the population described as high-risk [7] as there are many women without high-risk features, such as a family history of breast cancer, who could present a higher risk due to unhealthy lifestyles [1, 8].These programs should also not be limited to clinical settings as although breast cancer is usually discussed during consultations, communication between women and health workers may not always be effective, contributing to a lack of knowledge of this type of cancer among women [9].

There is growing evidence of the benefits of health promotion interventions for reducing cancer risk among high-risk individuals. It is essential that breast cancer health promotion programs are carried out in a more targeted, individualized way to ensure that they reach women [10]. Education improves people’s capability and reflective motivation; according to the Behaviour Change Wheel, these two determinants impact individuals’ intentions to engage in healthy behaviors [11]. Educational interventions are heterogeneous in terms of their design and content [12]. However, interventions based on theoretical models have been shown to be particularly effective [13]. Various channels have been used to convey messages to the public. Digital technology is particularly relevant as it has proven effective in breast cancer screening strategies [14] and among women with breast cancer [15]: webpages [16] are used as a form of communication between health professionals and patients, along with other forms of communication such as text messages, videos, or images [17].

The study’s main aim was to evaluate the preliminary effectiveness of an educational intervention using a web-app to improve knowledge of breast cancer risk factors and symptoms among women without breast cancer diagnosis in the Principado de Asturias (Spain). The secondary aim was the assessment of adherence to healthy eating and physical activity. The present study attempts to demonstrate that an educational intervention using a web-application based on the Behaviour Change Wheel model could be useful for improving knowledge of breast cancer risk factors and symptoms, as well as improving adherence to a healthy diet and physical activity among young women without a previous diagnosis of breast cancer.

## Materials and methods

### Design

A pragmatic randomized pilot trial was carried out from November 2019 to November 2020 at the University of Oviedo. The study was registered at ClinicalTrial.gov with number NCT04396665 and approved by the Principado de Asturias Ethics Committee (ref. 147/19). This study is reported following the CONSORT extension for pragmatic pilot trials [18].

### Participants and recruitment

Two hundred and eighty-five women aged 25–50 were recruited in December 2019. The selection was made by convenience from a cohort of a previous study. Inclusion criteria were not having a diagnosis of breast cancer, having access to the internet via a fixed or mobile device, and signing an informed consent form to voluntarily join the study.

The women were contacted by telephone to request their collaboration. The study objective and phases were explained to them during the telephone call. Those who verbally agreed to participate were sent an email with more detailed information and the informed consent form, which was completed online.

Twenty-five of the women who were contacted by telephone decided not to participate. The remaining 260 were grouped according to their place of residence: Avilés (*n* = 148) and Oviedo plus Gijón (*n* = 112). A number (1 and 2) was assigned to each cluster. Using Excel Aleat Function each number was assigned into the intervention group (IG = Avilés) and the control group (CG = Oviedo plus Gijón). Considering the potential bias and given the impossibility of blinding the intervention for participants, because those that got access to the web-app were able to recognize that they were in the intervention group, we opted for clustering the women to avoid any potential contamination that could result from the women exchanging information due to the proximity of their residences (the distance from Avilés to Oviedo and Gijon is about 35 km).

### Data collection and measurements

The primary endpoint with respect to preliminary effectiveness of the educational intervention was the improvement in the knowledge of breast cancer risk factors and symptoms from baseline to 6-month intervention. Additional analyses were done on the change in the adherence to healthy eating and physical activity.

The women who signed the informed consent form were sent the data collection form by email pre-intervention and again 6 months after the intervention (post-intervention). The form included five sociodemographic questions (age, marital status, level of education, weight, and height), the Motiva.Diaf questionnaire [19], and two dimensions included in the MARA questionnaire [20] relating to knowledge of breast cancer risk factors and identification of breast cancer symptoms (subscales reliability *α* = 0.74–0.92).

We used the two dimensions of the MARA questionnaire [20]. Nine risk factors and nine symptoms were included with responses coded dichotomously (is a factor/is not a factor – is a symptom/is not a symptom) as well as quantitatively for all risk factors and symptoms individually (correct responses average; range 0–9).

The Motiva.Diaf questionnaire [19] was used to assess adherence to dietary and physical activity recommendations. The questionnaire is divided into two sections and only 12 questions relating to behavioral recommendations were used (seven about diet and five about physical activity), clustered into one dimension. Questions were dichotomously coded according to the percentage of adherence (follows recommendation/does not follow recommendation) and quantitatively coded for all recommendations (mean number of recommendations followed by each woman; range 0–12).

### Intervention

Women in the IG participated in a 6-month intervention (January–June 2020) based on the Behaviour Change Wheel Model [11] in which education, persuasion, training, incentivization, and modeling were used to improve capability and motivation (Table 1). Women in the CG received the usual care.

**Table 1:** Sources of behavior, intervention functions, and needs included in the educational intervention

Needs targeted in dietary interventions	COM-B	Intervention functions
Knowledge regarding the recommended frequency and quantity of consumption of the different food groups	Psychological capability	Education
Acknowledging the positive effects of healthy eating on the disease	Psychological capability Reflective motivation	Education Persuasion
Knowledge regarding different techniques for preparing food in a healthy way	Psychological capability Automatic motivation	Education Incentivization

Needs targeted in physical activity interventions	COM-B	Intervention functions
Knowledge regarding duration, frequency, and appropriate ways to perform physical activity	Psychological capability	Education Persuasion
Designing routines and learning how to perform exercises	Psychological capability Physical capability Automatic motivation	Training Education Modeling Incentivization
Acknowledgment of the benefits of regular and safe physical activity	Psychological capability Reflective motivation	Education Persuasion

Needs related to breast cancer symptoms and risks	COM-B	Intervention functions
Knowledge regarding the signs, symptoms, and risk factors for breast cancer	Psychological capability Reflective motivation	Education Persuasion
Knowledge of breast examination techniques	Psychological capability Physical capability Automatic motivation	Training Education Modeling

A web-app specifically designed for this study was used. The app was divided into six sections: (i) breast cancer risk factors; (ii) early detection of breast cancer (breast self-examination and signs and symptoms); (iii) physical activity (‘create your own routine’ and related resources); (iv) nutrition; (v) news (on nutrition, physical activity, and evaluations); (vi) contests, where women could complete challenges related to nutrition and physical activity.

The content of sections (i) and (ii) was permanent from the beginning to the end of the intervention and consisted of texts and images relating to the risk factors and symptoms of breast cancer, as well as six explanatory videos showing the steps required to correctly perform a breast self-examination. Both sections were developed following the advice of a breast cancer nurse practitioner and a medical doctor.

Sections (iii) and (iv) included information in the form of texts, images, or videos related to physical activity and nutrition and were updated twice a week. In relation to physical activity, the women were provided with information about different outdoor walking routes in the Principado de Asturias. Throughout the intervention, they could access 58 videos showing a woman performing exercises of varying intensity and difficulty, arranged by body zones, which served as motivation and guidance enabling them to learn and perform them correctly. The videos were accompanied by an exercise plan (frequency, number of repetitions, and combination of exercises). These resources were intended to help participants design routines and activity plans to be carried out at home. In the nutrition section, women had access to healthy recipes and videos featuring a nutrition expert who gave them tips and general recommendations for a healthy diet. These were updated every 15 days and the expert was also available for questions about dietary habits via email. Sections iii and iv were developed according to the advice and support from a sports and nutrition technician, respectively.

Section (v) consisted of news concerning the topic of the study from the press or social media, which was of interest to the participants by the research team. This section was developed by a nurse and a lecturer and researcher in health promotion.

Finally, a contest was created in which participants were encouraged to complete the routines and recipes suggested in sections (iii) and (iv) and take a photograph of a routine while they were doing it or a photograph of a recipe they had prepared displaying a logo designed specifically for the intervention. They were then asked to share the photograph with the other women in the contest section. Each completed challenge gave the participant a score. At the beginning of the intervention, the women were informed that the participant with the highest score at the end of the intervention would receive a prize.

### Sample size

The sample size was calculated based on the MARA questionnaire scores. A sample size of 76 participants per group was required to detect an average difference between baseline and post-intervention measurements equal to or greater than 0.5 (SD 1.75) points in the total score of each of the two dimensions of the questionnaire with a two-sided significance level of 5%, a power of 80% and a correlation between the two measurements of 0.81 [20].

### Statistical analysis

Since the percentage of losses was significantly differential between groups and especially important in the intervention group (26.1%), it was considered more appropriate not to apply the intention-to-treat principle and a per protocol analysis was carried out [21].

Percentages, means, and standard deviations (SD) were calculated according to the nature of the variables in order to describe the characteristics of each individual in the sample. The assumption of normality was assessed using the Kolmogorov–Smirnov test and, if fulfilled, the corresponding parametric tests were carried out.

Chi-squared and McNemar’s tests were used to calculate differences between qualitative variables, percentage of breast cancer risk factors and symptoms identified and adherence to recommendations on diet and physical activity, in post-test between groups and change from pre-test to post-test, respectively. *t*-Student test was used to determine the impact of the intervention on the number of risk factors and symptoms identified. All analyses were performed using SPSS® (version 24.0) software from IBM®.

## Results

### Baseline characteristics of the population

**Fig. 1:**
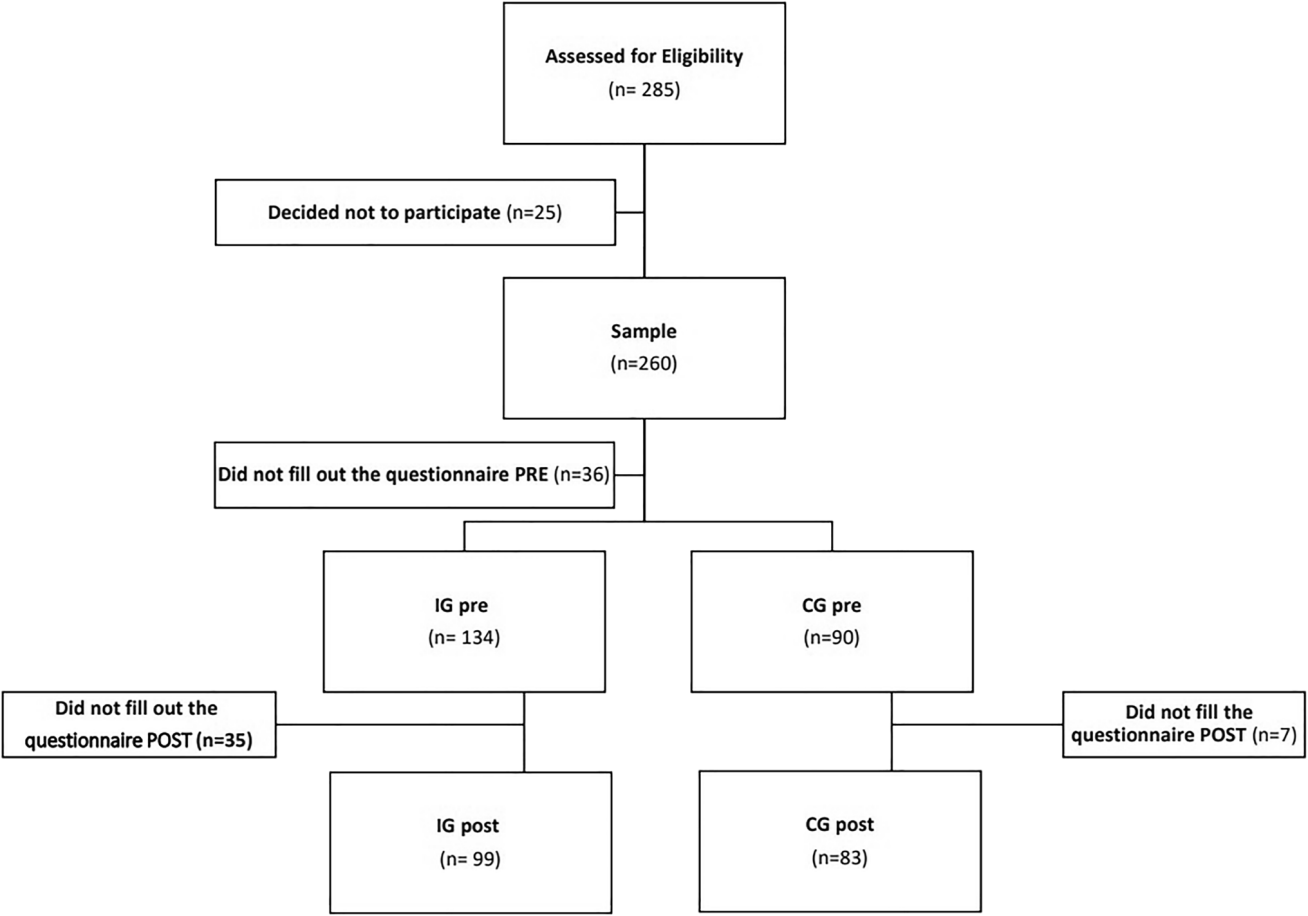
Flow diagram of participants

Of the 260 women recruited, 36 did not complete the baseline questionnaires and were excluded from the study. A total of 224 women, distributed across the IG (*n* = 134) and the CG (*n* = 90), participated in the intervention phase (Fig. 1). Their personal characteristics are described in Table 2. No significant differences in adherence to healthy recommendations or knowledge were found between the groups at the beginning of the study, confirming that the two populations were homogeneous (Table 2).

**Table 2:** Baseline characteristics of the sample (CG = 90; IG = 134)

	Total	CG	IG	*t*	*p*
Age, mean (SD)	39.51 (7.017)	38.18 (7.334)	40.39 (6.685)	2.058	0.041
BMI, mean (SD)	24.97 (4.3811)	23.90 (4.356)	24.67 (4.387)	1.253	0.211
Risk factors identified, mean (SD)	4.17 (1.834)	4.24 (1.644)	4.13 (1.956)	0.470	0.639
Symptoms identified, mean (SD)	6.00 (1.753)	6.12 (1.483)	5.91 (1.913)	0.949	0.344

	Total	CG	IG	*χ*^2^	*p*
Marital status %					
Single, divorced, or widowed	44.3	49.4	40.7	2.478	0.253
Married or with a partner	55.7	50.6	59.3		
Level of education					
No higher education	33.3	26.4	38.2	3.179	0.075
Higher education	66.7	73.6	61.8		
Dietary recommendations, % adherence					
Daily consumption of 4 to 6 servings of the following foods: bread, grains, pasta, rice, and potatoes (Q1)	62.2	65.6	71.6	0.936	0.333
Daily consumption of ≥ 3 servings of fresh fruit (Q2)	70.5	73.3	68.7	0.567	0.452
Daily consumption of ≥ 2 servings of vegetables (Q3)	80.8	81.1	80.6	0.009	0.924
Daily consumption of 2 to 4 servings of milk and dairy products (Q4)	85.3	82.2	87.3	1.111	0.292
Weekly consumption of 3 to 4 servings of fish (Q5)	58.0	52.2	61.9	2.088	0.148
Weekly consumption of 3 to 4 servings of low-fat meats (Q6)	81.7	81.1	82.1	0.034	0.853
Weekly consumption of 3 to 7 servings of nuts (Q7)	52.2	52.2	52.2	0.000	0.998
Physical activity recommendations, % adherence					
Walking at least 30 min a day (Q8)	68.3	62.2	72.4	2.570	0.109
Using stairs instead of elevators or escalators (Q9)	65.2	60.0	68.7	1.778	0.182
Walking instead of using means of transport for short distances (Q10)	86.6	80.0	91.0	5.662	0.017
Lightly moving after eating instead of resting (Q11)	29.5	31.1	28.4	0.196	0.658
Moving every 30 min while sedentary (Q12)	25.9	21.1	29.1	1.793	0.181
Identification of breast cancer risk factors, % of correct responses					
Use of hormone replacement therapy	46.4	48.9	44.8	0.366	0.545
Menarche before the age of 12 years	12.5	12.2	12.7	0.011	0.918
Menopause after the age of 55	10.7	10.0	11.2	0.080	0.777
Infertility/Nulliparity	15.6	13.3	17.2	0.599	0.439
First child before the age of 30	66.1	63.3	67.9	0.503	0.478
High fat diet	76.8	78.9	75.4	0.373	0.541
Overweightness	70.1	72.2	68.7	0.326	0.568
Smoking	87.5	92.2	84.3	3.067	0.080
Excessive alcohol intake/day	31.7	33.3	30.6	0.186	0.666
Identification of breast cancer symptoms, % of correct responses					
Nipple discharge (fluid or blood)	83.9	85.6	82.8	0.295	0.587
Breast and/or armpit swelling	85.3	86.7	84.3	0.234	0.628
Changes in the size, shape, or appearance of the breast or nipple	91.1	90.0	91.8	0.212	0.645
Pain in a breast or armpit	59.4	63.3	56.7	0.977	0.323
Sensation of nipple tightness	37.5	34.4	39.6	0.599	0.439
Lump or thickening under the armpit	92.4	96.7	89.6	3.886	0.049
Stretch marks on one or both breasts	5.4	5.6	5.2	0.012	0.914
Dimple or puckering in the skin of the breast	52.7	53.3	52.2	0.026	0.872
Lump or thickening of the breast	91.1	96.7	87.3	5.792	0.016

Forty-two women withdrew from the study between the pre-intervention and the post-intervention, 35 from the IG and seven from the CG, as all of them (100%) failed to complete the post-intervention questionnaires (Fig. 1).

### Breast cancer risk factor and symptom identification

The mean number of risk factors and symptoms identified was statistically significantly higher among women in the IG (risk factors 5.19, SD = 2.093; symptoms 7.09, SD = 1.179) compared to women in the CG (risk factors 4.33, SD = 1.668; symptoms 6.33, SD = 1.570) after the intervention with a moderate to higher association (Table 3). Furthermore, the number of correct responses from pre to post was statistically significant for women in the IG, but not for those in the CG (Table 3).

**Table 3:** Mean number (SD) of breast cancer risk factors and symptoms correctly identified after the intervention and mean change from pre- to post-intervention (*n* = 83) and IG (*n* = 99)

	Post-intervention					Change from pre to post			
	Total	CG	IG	*p*	*d-*Cohen	CG	*p*	IG	*p*
Mean number of risk factors identified (SD)	4.80 (1.954)	4.33 (1.668)	5.19 (2.093)	< 0.001	0.476	0.09	0.568	1.06	< 0.001
Mean number of symptoms identified (SD)	6.74 (1.420)	6.33 (1.570)	7.09 (1.179)	< 0.001	0.676	0.21	0.211	1.18	< 0.001

Women in the IG identified a higher percentage of breast cancer risk factors post-intervention, with significant percentages for 3 of the 9 risk factors. Furthermore, the percentage of breast cancer risk factors identified improved among the women in the IG from pre-intervention to post-intervention for 6 risk factors versus 1 risk factor in the CG (Table 4).

**Table 4:** Effects of the intervention on breast cancer risk factors and symptoms

Risk factors	Post-intervention					% of change from pre to post			
	Total	CG	IG	*χ*^2^	*p*	CG	*p*	IG	*p*
Use of hormone replacement therapy	45.1	41.0	48.5	1.032	0.310	− 7.9	0.405	+ 3.7	0.845
Menarche before the age of 12 years	21.4	14.5	27.3	4.404	0.036	+ 2.3	0.774	+ 14.6	0.004
Menopause after the age of 55	17.0	8.4	24.2	7.984	0.005	− 1.6	1.000	+ 13.0	0.017
Infertility/Nulliparity	23.6	18.1	28.3	2.609	0.106	+ 5.8	0.388	+ 11.1	0.021
First child before the age of 30	63.7	49.4	75.8	13.573	< 0.001	− 13.9	0.099	+ 7.9	0.281
High fat diet	86.8	84.3	88.9	0.817	0.366	+ 5.4	0.424	+ 13.5	0.023
Overweightness	88.5	86.7	89.9	0.439	0.507	+ 14.5	0.019	+ 21.2	< 0.001
Smoking	95.1	94.0	96.0	0.378	0.539	+ 1.8	0.754	+ 11.7	0.002
Excessive alcohol intake/day	38.5	36.1	40.4	0.346	0.556	+ 2.8	0.832	+ 9.8	0.265

Symptoms	Post-intervention					% of change from pre to post			
	Total	CG	IG	*χ*^2^	*p*	CG	*p*	IG	*p*
Nipple discharge (fluid or blood)	94.0	89.2	98.0	6.189	0.013	+ 3.6	0.774	+ 15.2	< 0.001
Breast and/or armpit swelling	90.7	85.5	94.9	4.718	0.030	− 1.2	1.000	+ 10.6	0.008
Changes in the size, shape, or appearance of the breast or nipple	96.2	92.8	99.0	4.721	0.030	+ 2.8	0.754	+ 7.2	0.008
Pain in a breast or armpit	67.0	62.7	70.7	1.326	0.250	− 0.6	1.000	+ 14.0	0.020
Sensation of nipple tightness	59.9	44.6	72.7	14.892	< 0.001	+ 10.2	0.108	+ 33.1	< 0.001
Lump or thickening under the armpit	97.3	96.4	98.0	0.429	0.512	− 0.3	1.000	+ 8.4	0.070
Stretch marks on one or both breasts	8.8	7.2	10.1	0.464	0.496	+ 1.6	1.000	+ 4.9	0.180
Dimple or puckering in the skin of the breast	64.8	60.2	68.7	1.413	0.235	+ 6.9	0.359	+ 16.5	0.001
Lump or thickening of the breast	95.6	94.0	97.0	0.963	0.326	− 2.7	0.727	+ 9.7	0.109

Percentage of breast cancer risk factors and symptoms identified among the women in post-intervention and difference from pre-intervention to post-intervention for CG (*n* = 83) and IG (*n* = 99) individually

With regard to the identification of breast cancer symptoms post-intervention, the percentage was higher among women in the IG, especially for 5 symptoms. Considering each group separately, the percentage of symptoms identified improved significantly in the IG for 6 of the symptoms between pre-intervention and post-intervention, an improvement not observed in the CG (Table 4).

### Healthy dietary and physical activity behaviors

Positive statistically significant differences were observed in the IG after the educational intervention for 4 of the intervention recommendations, 1 relating to diet and 3 relating to physical activity. Meanwhile, adherence to 8 of the 12 recommendations significantly improved among participants in the IG from pre-intervention to post-intervention (Table 5).

**Table 5:** Percentage of adherence to recommendations on diet and physical activity post-intervention and difference between pre-intervention and post-intervention for the CG (*n* = 83) and IG (*n* = 99)

	Post-Intervention					% of change from pre to post			
	Total	CG	IG	*χ*^2^	*p*	CG	*p*	IG	*p*
Dietary behaviors									
Q1	80.8	67.5	91.9	17.375	< 0.001	+ 1.8	0.856	+ 20.3	0.002
Q2	78.6	73.5	82.8	2.336	0.126	+ 0.2	1.000	+ 14.1	0.050
Q3	89.6	86.7	91.9	1.292	0.256	+ 5.6	0.289	+ 11.3	0.013
Q4	86.3	85.5	86.9	0.067	0.796	+ 3.3	1.000	-0.4	1.000
Q5	68.7	67.5	69.7	0.104	0.747	+ 15.3	0.002	+ 7.8	0.383
Q6	87.4	85.5	88.9	0.458	0.499	+ 4.4	0.629	+ 6.8	0.039
Q7	64.3	61.4	66.7	0.536	0.464	+ 9.2	0.210	+ 14.5	0.002
Physical activity behaviors									
Q8	86.3	80.7	90.9	3.953	0.047	+ 18.5	< 0.001	+ 18.5	< 0.001
Q9	76.9	69.9	82.8	4.264	0.039	+ 9.0	0.078	+ 14.1	0.004
Q10	88.5	88.0	88.9	0.039	0.844	+ 8.0	0.092	-2.1	0.508
Q11	45.1	32.5	55.6	9.669	0.002	+ 1.4	0.832	+ 27.4	< 0.001
Q12	35.7	28.9	41.4	3.072	0.080	+ 7.8	0.286	+ 12.3	0.215

The number of recommendations followed increased in both groups after the intervention, with a mean of 8.28 (SD = 2.265) for the CG, and a mean of 9.38 (SD = 1.789) for the IG. Differences in this respect were not significant between the groups.

## Discussion

The results of our study confirm that a web-app-based educational intervention can improve the level of adherence to dietary and physical activity, in 8 of 12 recommendations, as well as the level of knowledge, improved from pre-intervention to post-intervention for 6 risk factors and 6 symptoms, among women without a breast cancer diagnosis aged 25–50.

There are two relevant aspects to consider in the design of the intervention. On the one hand, the active role granted to the participants. Various researchers suggest that breast cancer prevention policies should include strategies involving women in developing healthy lifestyles [22]. On the other hand, making women aware of their specific risks is essential to improve the uptake of healthy behaviors [23].

The content of the web-app was similar to the one employed in previous interventions using webpages [8] and other media [24, 25] and similar objectives, especially regarding information about risk factors and primary prevention activities. According to various authors, it is essential to include this content when aiming to reduce the incidence of cases, as this content can be useful for the general population and not only for those considered at risk [8].

Satisfactory outcomes have also been observed among women participating in interventions facilitating knowledge acquisition, as well as in previous studies using a web-app as a medium to disseminate information about cancer prevention [26].

After the intervention, women in the IG identified a higher number of risk factors than before the intervention than women in the CG. Awareness of risk factors is essential to enable women to take an active role in eliminating them [6] through self-examination [13]. To this end, health promotion strategies must be developed for women [27] so they can learn and become aware of the risk factors involved and try to diminish them [28]. Previous studies show that women are in favor of learning about risk factors and are interested in modifying them once identified [29]. However, it must be stressed that non-modifiable risk factors were the most difficult to identify. This corroborates the findings of Livaudais-Toman et al. [24], where early menarche and late menopause were two of the most difficult risk factors to identify by the women in the study. While this cannot be considered positive for risk estimation purposes, it is less relevant from an interventionist point of view as these factors cannot be changed. Therefore, helping women to identify their individual risk factors for breast cancer effectively allows them to estimate their risk, become aware of their situation, and adopt preventive measures. Rainey et al. [30] highlighted the importance of creating educational materials and providing individualized advice to help women make breast cancer prevention decisions.

Similarly, women in the IG significantly improved their knowledge of breast cancer symptoms. Knowledge of these symptoms encourages early identification, allowing for immediate effective diagnostic tests and early treatment if needed, which is related to improved disease progression [1]. Symptoms related to changes in breast appearance were the most difficult to identify by both groups. Similar results were observed by Qasim et al. [31]. This may be due to information campaigns focusing on the presence of lumps and downplaying the importance of other symptoms that are nonspecific to breast cancer. The results suggest that more attention needs to be paid to identifying these symptoms.

An improvement in the behavior of both groups was observed, although it was only significant in the IG. Physical activity and a healthy diet have a positive and clearly established impact on breast cancer. The risk of developing breast cancer decreases in women who engage in regular physical activity [32] and in those who have healthy dietary habits [33, 34]. It is therefore essential to address these behaviors when developing breast cancer prevention strategies. As suggested by Cadmus-Bertram et al. [35], who developed a specific intervention including these two behaviors targeting women at high-risk of developing breast cancer. In the same vein, a systematic review by Cathcart-Rake et al. [36] suggests that physical activity, lower consumption of fatty meats, and higher consumption of vegetables reduce the risk of developing breast cancer among young women in particular.

Therefore, an intervention based on the use of a web-app such as the one described in this study can achieve an increase in knowledge of the disease as well as behavioral changes. In view of the results obtained using the web-app the next step could be to regularly offer this intervention as a supplementary resource for all women to address some of the clinical challenges faced by health services in preventing breast cancer [6].

The strengths of this study include the alignment of its objectives with one of the priorities identified by the European Commission [37]. For example, the incorporation of digital technology into the field of health and its potential benefits. In addition, the design of the web-app enables women to actively participate in their own breast cancer prevention in an individualized way, as they can identify their own level of risk and the behaviors they wish to or are able to change.

The limitations of this study include those inherent to any study of this kind. Although the results point to an estimate of a real-life implementation, it is still necessary to conduct randomized experimental studies with a larger population to compare the results. The online format of the intervention and the self-reporting of information by participants may have introduced obsequiousness bias in relation to behavioral variables, including the impossibility of verifying that it was indeed the participant who was completing the questionnaires. However, considering the nature of the questions and the relevance of breast cancer as a health problem affecting many women, we believe that these limitations may not be significant. This study had a 30% drop out rate, what could be considered as bias. Nonetheless, even the causes were not assessed, is important to highlight that the intervention was carried out during initial phase of COVID pandemic, and we hypothesize that it could explain both why the post-test intervention was minor than the pre- intervention participation (before COVID).

## Conclusion

The preliminary results of this study suggest that the educational intervention, using a web-application based on the Behaviour Change Wheel model, was useful for improving knowledge of breast cancer risk factors and symptoms, as well as improving adherence to a healthy diet and physical activity among women without a previous diagnosis of breast cancer. Nonetheless, randomized controlled trials should be conducted in the future to provide more rigorous evidence of the effects of educational interventions based on the use of web-apps in enhancing early detection and prevention of breast cancer.

## Supplementary Information

Below is the link to the electronic supplementary material.

Supplementary file1 (DOCX 14 KB)

Supplementary file2 (DOCX 19 KB)

## Data Availability

Research data are no shared.

## Code availability

Not aplicable.
