# Supplementary material for: Use a web-app to improve breast cancer risk factors and symptoms knowledge and adherence to healthy diet and physical activity in women without breast cancer diagnosis (Precam project)
Source: Cancer Causes Control. 2022 Nov 8;34(2):113–22. doi: 10.1007/s10552-022-01647-x (PMC9877062; doi:10.1007/s10552-022-01647-x)
Supplement: Supplementary file 1 — Supplementary file1 (DOCX 14 KB) [file 10552_2022_1647_MOESM1_ESM.docx]

| **Questions included in table 5** | |
| --- | --- |
| **Dietary behaviors** | |
| Q1 | A healthy diet includes a daily consumption of 4-6 servings of the following foods: bread, grains, pasta, rice, and potatoes. A service of pasta or rice is generally a plate, a serving of grains is generally a bowl, a serving of pan is generally 3 or 4 slices or a role (40- 60 grams) and a serving of potatoes is generally 1 large or 2 small potatoes (150- 200 grams). |
| Q2 | A healthy diet includes the daily consumption of 3 or more servings of fresh fruit. An example of a serving would be a medium piece, a cup of cherries, two slices of melon... (A serving equals 120 - 200 grams) |
| Q3 | A healthy diet includes the daily consumption of 2 or more servings of vegetables, raw or cooked without fat. A serving of these foods would be, for example: a plate of salad, a plate of cooked vegetables, 1 large tomato, 2 carrots... (A serving equals 150- 200 grams) |
| Q4 | A healthy diet includes the daily consumption of 2 to 4 servings of milk and dairy products. A serving is considered to be, a glass of milk (200-250 ml), 2 yogurts (200-250 grams), or 3 slices of cheese (40-60 grams of cured cheese or 80-125 grams of fresh cheese) |
| Q5 | A healthy diet includes weekly consumption of 3 to 4 servings of fish, or the equivalent of one individual fillet (125-150 grams) |
| Q6 | A healthy diet includes the weekly consumption of 3 to 4 servings of meat low in fat, with no visible fat, and with no skin on fowl. A serving is a small fillet, or a quarter chicken or rabbit (125- 150 grams) |
| Q7 | A healthy diet consists of a weekly consumption of 3 to 7 servings of nuts. One serving equals a handful (20-30 grams) |
| **Physical activity behaviors** | |
| Q8 | Walking at least 30 minutes per day is considered healthy physical activity |
| Q9 | Using the stairs instead of the elevator or escalator is considered healthy physical activity |
| Q10 | Walking instead of using transport methods for short distances is considered healthy physical activity |
| Q11 | Lightly moving (taking a stroll for example…) after eating instead of resting (for example, sitting or lying down...) is considered healthy physical activity |
| Q12 | Moving (for example, getting up and walking or getting up and stretching for 2 to 3 minutes) every 30 minutes while being sedentary (for example, watching TV or working...) is considered healthy physical activity |
